# Analytical validation and implementation of a pan cancer next-generation sequencing panel, CANSeq^TM^Kids for molecular profiling of childhood malignancies

**DOI:** 10.3389/fgene.2023.1067457

**Published:** 2023-02-09

**Authors:** Kala F. Schilter, Brandon A. Smith, Qian Nie, Kathryn Stoll, Juan C. Felix, Jason A. Jarzembowski, Honey V. Reddi

**Affiliations:** ^1^ Precision Medicine Laboratory, Department of Pathology, Medical College of Wisconsin, Milwaukee, WI, United States; ^2^ Pathology and Laboratory Medicine, Medical College of Wisconsin, Milwaukee, WI, United States

**Keywords:** pan cancer assay, childhood cancer, next generation sequencing panel, molecular profiling, clinical implementation, assay validation

## Abstract

Next-Generation Sequencing (NGS) allows rapid analysis of multiple genes for the detection of clinically actionable variants. This study reports the analytical validation of a targeted pan cancer NGS panel CANSeq^TM^Kids for molecular profiling of childhood malignancies. Analytical validation included DNA and RNA extracted from de-identified clinical specimens including formalin fixed paraffin embedded (FFPE) tissue, bone marrow and whole blood as well as commercially available reference materials. The DNA component of the panel evaluates 130 genes for the detection of single nucleotide variants (SNVs), Insertion and Deletions (INDELs), and 91 genes for fusion variants associated with childhood malignancies. Conditions were optimized to use as low as 20% neoplastic content with 5 ng of nucleic acid input. Evaluation of the data determined greater than 99% accuracy, sensitivity, repeatability, and reproducibility. The limit of detection was established to be 5% allele fraction for SNVs and INDELs, 5 copies for gene amplifications and 1,100 reads for gene fusions. Assay efficiency was improved by automation of library preparation. In conclusion, the CANSeq^TM^Kids allows for the comprehensive molecular profiling of childhood malignancies from different specimen sources with high quality and fast turnaround time.

## 1 Introduction

Childhood cancers including leukemias, and tumors of the central nervous system and renal tumors are the leading disease-related causes of death in children in the United States (Siegel et al., 2018). General treatment of childhood malignancies is a combination of surgery, cytotoxic chemotherapy, and radiotherapy, with long term side effects (Kopp et al., 2012). The discovery of more personalized and less harmful therapies is a rising need, however, childhood cancers currently represent less than 1% of new cancer diagnosis (Siegel et al., 2014). Evidence demonstrates that the frequency, distribution, and types of genetic alterations of childhood cancer may differ from adult tumors (Vogelstein et al., 2013), demanding the need for a better understanding of the molecular landscape of childhood malignancies.

Molecular profiling for childhood cancer usually comes into play after diagnosis or failure to respond to standard therapy. Profiling studies using next-generation sequencing (NGS) have facilitated widespread investigation of the molecular landscape of childhood cancers in the recent years leading to the identification of a large number of biomarkers across multiple childhood cancers with both small mutations and copy number variants (Grobner et al., 2018; Ma et al., 2018). Specifically, 17% of driver genes were mutated in both leukemias and solid tumors. *CDKN2A*, *IKZF1*, *ETV6*, *RUNX1*, and *FLT3* were the top genes mutated in leukemias, while somatic alterations in *ALK*, *NF1*, and *PTEN* primarily occurred in solid tumors, suggesting that the driver alterations are either common to cancer (e.g., cell cycle) or specific to pediatric cancer histotype (Ma et al., 2018). Given the uniqueness of childhood cancers, it is important to have a molecular profiling assay that is comprehensive and applicable across most if not all childhood malignancies.

In this study, we report the analytical validation of the CANSeq^TM^Kids assay which uses a targeted NGS panel that interrogates both DNA and RNA to provide comprehensive genomic information across 203 unique genes known to be associated with childhood malignancies. The assay was validated across multiple specimen types including fixed paraffin embedded (FFPE) tissue, cell blocks, blood, and bone marrow prior to clinical implementation for the evaluation of pediatric tumors.

## 2 Materials and methods

### 2.1 Panel content

CANSeq^TM^Kids is a comprehensive molecular profiling assay that evaluates relevant DNA mutations (SNVs, indels and CNVs) across 130 key genes and RNA fusions across 91 fusion transcript driver genes associated with pediatric cancer, in a single NGS assay (Table 1).

**TABLE 1:** Panel content (203 unique genes).

Hotspot genes		Copy number genes	Full length genes				Gene fusions		
*ABL1*	*FBXW7*	*NCOR2*	*ABL2*		*APC*	*RUNX1*	*ABL1*	*KMT2C*	*PAX5*
*ABL2*	*FGFR1*	*NOTCH1*	*ALK*		*ARID1A*	*SMARCA4*	*ABL2*	*KMT2D*	*PAX7*
*ALK*	*FGFR2*	*NPM1*	*BRAF*		*ARID1B*	*SMARCB1*	*AFF3*	*LM O 2*	*PDGFB*
*ACVR1*	*FGFR3*	*NRAS*	*CCND1*		*ATRX*	*SOCS2*	*ALK*	*MAML2*	*PDGFRA*
*AKT1*	*FLT3*	*NT5C2*	*CDK4*		*CDKN2A*	*SUFU*	*BCL11B*	*MAN2B1*	*PDGFRB*
*ASXL1*	*GATA2*	*PAX5*	*CDK6*		*CDKN2B*	*SUZ12*	*BCOR*	*MECOM*	*PLAG1*
*ASXL2*	*GNA11*	*PDGFRA*	*EGFR*		*CEBPA*	*TCF3*	*BCR*	*MEF2D*	*RAF1*
*BRAF*	*GNAQ*	*PDGFRB*	*ERBB2*		*CHD7*	*TET2*	*BRAF*	*MET*	*RANBP17*
*CALR*	*H3F3A*	*PIK3CA*	*ERBB3*		*CRLF1*	*TP53*	*CAMTA1*	*MKL1*	*RARA*
*CBL*	*HDAC9*	*PIK3R1*	*FGFR1*		*DDX3X*	*TSC1*	*CCND1*	*MLLT10*	*RECK*
*CCND1*	*HIST1H3B*	*PPM1D*	*FGFR2*		*DICER1*	*TSC2*	*CIC*	*MN1*	*RELA*
*CCND3*	*HRAS*	*PTPN11*	*FGFR3*		*EBF1*	*WHSC1*	*CREBBP*	*MYB*	*RET*
*CCR5*	*IDH1*	*RAF1*	*FGFR4*		*EED*	*WT1*	*CRLF2*	*MYBL1*	*ROS1*
*CDK4*	*IDH2*	*RET*	*GLI1*		*FAS*	*XIAP*	*CSF1R*	*MYH11*	*RUNX1*
*CIC*	*IL7R*	*RHOA*	*GLI2*		*GATA1*		*DUSP22*	*MYH9*	*SS18*
*CREBBP*	*JAK1*	*SETBP1*	*IGF1R*		*GATA3*		*EGFR*	*NCOA2*	*SSBP2*
*CRLF2*	*JAK2*	*SETD2*	*JAK1*		*GNA13*		*ETV6*	*NCOR1*	*STAG2*
*CSF1R*	*JAK3*	*SH2B3*	*JAK2*		*ID3*		*EWSR1*	*NOTCH1*	*STAT6*
*CSF3R*	*KDM4C*	*SH2D1A*	*JAK3*		*IKZF1*		*FGFR1*	*NOTCH2*	*TAL1*
*CTNNB1*	*KDR*	*SMO*	*KIT*		*KDM6A*		*FGFR2*	*NOTCH4*	*TCF3*
*DAXX*	*KIT*	*STAT3*	*KRAS*		*KMT2D*		*FGFR3*	*NPM1*	*TFE3*
*DNMT3A*	*KRAS*	*STAT5B*	*MDM2*		*MYOD1*		*FLT3*	*NR4A3*	*TP63*
*EGFR*	*MAP2K1*	*TERT*	*MDM4*		*NF1*		*FOSB*	*NTRK1*	*TSLP*
*EP300*	*MAP2K2*	*TPMT*	*MET*		*NF2*		*FUS*	*NTRK2*	*TSPAN4*
*ERBB2*	*MET*	*USP7*	*MYC*		*PHF6*		*GLI1*	*NTRK3*	*UBTF*
*ERBB3*	*MPL*	*ZMYM3*	*MYCN*		*PRPS1*		*GLIS2*	*NUP214*	*USP6*
*ERBB4*	*MSH6*		*PDGFRA*		*PSMB5*		*HMGA2*	*NUP98*	*WHSC1*
*ESR1*	*MTOR*		*PIK3CA*		*PTCH1*		*JAK2*	*NUTM1*	*YAP1*
*EZH2*	*MYC*				*PTEN*		*KAT6A*	*NUTM2B*	*ZMYND11*
*FASLG*	*MYCN*				*RB1*		*KMT2A*	*PAX3*	*ZNF384*
							*KMT2B*		

### 2.2 Sample cohort

A total of 65 samples including FFPE tissue (n = 32), cell blocks (n = 2), whole blood (n = 8), bone marrow (n = 4), cell lines (n = 7) and commercial controls (n = 12) were used in the validation (Table 2). The size of the sample cohort was established based on recommended guidelines (Jennings et al., 2017). This study was performed using retrospective specimens with known molecular profiling results, known diagnoses and represented different tumor types (Table 3). Specimens were de-identified per IRB guidelines prior to inclusion in the study. Due to the diverse nature of childhood cancers, the CANSeq^TM^Kids panel has been designed to evaluate both solid tumors and hematological tissues. An analytical validation plan outlining sample cohort, validation strategy and processes involved, was reviewed and approved prior to study start. This study was approved by the Medical College of Wisconsin Institutional Review Board.

**TABLE 2:** Study Cohort. Summary of clinical specimens and commercial controls used in study (n = 65).

Sample source	No. of samples	FFPE (n = 32)	Cell blocks (n = 2)	Whole blood (n = 8)	Bone marrow (n = 4)	Cell lines (n = 7)	Commercial controls (n = 12)
DNA only	23	5	0	2	4	5	7
RNA only	16	1	2	6	0	2	5
DNA & RNA	26	26	0	0	0	0	0

**TABLE 3:** Study Cohort. Details of specimens used in study.

Sample ID	Diagnosis	Neoplastic content	Nucleic acid
FFPE tissue (n = 32)			
P-Validation 1	anaplastic large cell lymphoma	80%	DNA & RNA
P-Validation 2	inflammatory myofibroblastic tumor	80%	DNA & RNA
P-Validation 3	CIC-translocation sarcoma	20%	DNA & RNA
P-Validation 4	CIC-translocation sarcoma	100%	DNA & RNA
P-Validation 5	giant cell fibroblastoma	100%	DNA & RNA
P-Validation 6	mucoepidermoid carcinoma	40%	DNA & RNA
P-Validation 8	cellular mesoblastic nephroma	100%	DNA & RNA
P-Validation 10	Ewing sarcoma	100%	DNA & RNA
P-Validation 12	Ewing sarcoma	100%	DNA & RNA
P-Validation 13	desmoplastic small round cell tumor	100%	DNA & RNA
P-Validation 15	alveolar rhabdomyosarcoma	80%	DNA
P-Validation 17	low-grade fibromyxoid sarcoma	90%	DNA & RNA
P-Validation 18	low-grade fibromyxoid sarcoma	100%	DNA & RNA
P-Validation 19	diffuse large B-cell lymphoma	100%	DNA & RNA
P-Validation 20	myeloid sarcoma	98%	DNA & RNA
P-Validation 21	pilocytic astrocytoma	60%	DNA & RNA
P-Validation 22	pilocytic astrocytoma	100%	DNA & RNA
P-Validation 23	B-ALL	98%	DNA & RNA
P-Validation 24	double-hit lymphoma	100%	DNA & RNA
P-Validation 25	high-grade B-cell lymphoma	100%	DNA & RNA
P-Validation 26	lipoblastoma	20%	DNA & RNA
P-Validation 27	lipoblastoma	5%	DNA & RNA
P-Validation 28	ependymoma	100%	DNA & RNA
P-Validation 29	synovial sarcoma	100%	DNA & RNA
P-Validation 30	synovial sarcoma	100%	DNA & RNA
P-Validation 31	alveolar soft part sarcoma	100%	DNA & RNA
P-Validation 32	aneurysmal bone cyst	100%	DNA & RNA
P-Validation 33	Glioblastoma with biphasic morphology	95%	DNA
P-Validation 34	Optic Nerve Tumor	95%	DNA
P-Validation 35	Cystic botryoid rhabdomyosarcoma	75%	DNA
P-Validation 36	Round cell malignant neoplasm	50%	DNA
P-Validation 39	Likely NSCLC	30%	RNA
Cell Blocks (n = 2)			
P-Validation 37	Likely NSCLC	20%	RNA
P-Validation 38	Likely NSCLC	15%	RNA
Whole Blood (n = 8)			
M_Validation_07	AML Unspecified	N/A	DNA
M_Validation_11	AML Unspecified	N/A	DNA
M_Validation_22	AML Unspecified	N/A	RNA
M_Validation_23	AML w/MLL	N/A	RNA
M_Validation_24	APL	N/A	RNA
M_Validation_26	ALL	N/A	RNA
M_Validation_33	CML	N/A	RNA
M_Validation_37	AML Unspecified	N/A	RNA
Bone Marrow (n = 4)			
M_Validation_01	Pancytopenia	N/A	DNA
M_Validation_03	AML Unspecified	N/A	DNA
M_Validation_10	AML Unspecified	N/A	DNA
M_Validation_16	AML Unspecified	N/A	DNA
Cell Lines (n = 7)			
Coriell cell line NA12878	HapMap lymphoblastoid cell line	N/A	DNA
Coriell cell line NA18507	HapMap lymphoblastoid cell line	N/A	DNA
Coriell cell line NA19240	HapMap lymphoblastoid cell line	N/A	DNA
Cell line RKO	Colon Carcinoma cell line	N/A	DNA
Cell line NCI-H1650	Lung Adenocarcinoma cell line	N/A	DNA
Cell line NCI-H2228	Lung Adenocarcinoma cell line	N/A	RNA
Cell line LC2/AD	Lung Adenocarcinoma cell line	N/A	RNA
Commercial Contrived Controls (n = 12)			
AcroMetrix Oncology Hotspot Control8 (AOHC)	N/A; catalog # 969056	N/A	DNA
Seraseq Tri Level DNA Mutation Mix Control	N/A; catalog # 0710–0097	N/A	DNA
SeraCare normal colon RNA	N/A; catalog # AM7986	N/A	RNA
SeraCare normal lung RNA	N/A; catalog # AM7968	N/A	RNA
Seraseq Fusion RNA Mix v3	N/A; catalog # 0710–0431	N/A	RNA
Seraseq FFPE NTRK Fusion RNA Reference Material	N/A; catalog # 0710–1,031	N/A	RNA
Seraseq Lung/Brain CNV Mix (x3)	N/A; catalog # 0710–0415	N/A	DNA
Seraseq Tumor Mutation DNA Mix v2	N/A; catalog # 0710–0095	N/A	DNA
AcroMetrix Hotspot DNA Ladder	N/A; catalog # 10026229	N/A	DNA
AV Master CNV (x4)	N/A; supplied by ThermoFisher	N/A	DNA
AV Master Hotspot	N/A; supplied by ThermoFisher	N/A	DNA
AV Master Fusion	N/A; supplied by ThermoFisher	N/A	RNA

### 2.3 DNA and RNA extraction

DNA and RNA from all specimens was extracted per established protocols. FFPE specimens were macro dissection-enriched prior to extraction. DNA quantification and quality was evaluated using the NanoDrop 2000 (Thermo Fisher Scientific, Waltham, MA) and considered acceptable if the resultant A260/A280 absorbance ratio was between 1.8 and 2.1. RNA quantification was evaluated using the Qubit 2.0 Fluorometer (Thermo Fisher Scientific, Waltham, MA) and was considered acceptable if sufficient quantity of RNA to ensure a 10 ng input was obtained for downstream processing.

### 2.4 Library preparation, templating and sequencing

Libraries were prepared by both manual and automated Ion Chef process. For the DNA portion of library preparation, the manual library preparation requires 8 µL with a concentration of 2.5 ng/μL whereas the automated library preparation requires 15 µL with a concentration of 0.7 ng/μL. The RNA requirements are slightly less with 5 µL with a concentration of 2 ng/μL for manual prep and 10 µL with a concentration of 1 ng/μL for the automated process. The manual process followed the Oncomine™ Childhood Cancer Research Assay (OCCRA) (Thermo Scientific, Waltham, MA) and the Ion AmpliSeq™ Library Preparation user guide. The Automated library preparation used the Oncomine™ Childhood Cancer Research Assay, Chef-Ready kit on the Ion Chef (Thermo Fisher Scientific). Libraries were barcoded with IonCode™ Barcode Adapters 1–384 Kit and normalized to 100 pmol/L by the Equalizer kit (Thermo Scientific, Waltham, MA). DNA and RNA libraries were then combined and diluted at an 80:20 DNA:RNA ratio at ∼50p.m. and templated overnight on the Ion 540 chip using Ion 540™ Kit—Chef (Thermo Scientific, Waltham, MA).

Sequencing was performed using 540 chips on the Ion GeneStudio™ S5 Prime Sequencer (Thermo Fisher Scientific, Waltham, MA). Raw reads from sequencing were processed and aligned to the reference genome hg19 on Ion Torrent Suite Software versions 5.12 and 5.14 (Thermo Fisher Scientific, Waltham, MA) and the run metrics of the Ion Torrent Suite used to determine quality control of sequencing runs. The minimum cutoff of ISP (Ion Sphere™ Particle) loading was 80% and the maximum of polyclonal ISPs was 50%, with threshold for total reads at 60M. The minimum percent usable reads were set to be 30%, and the minimum raw accuracy was 99%.

Variant calling and fusion detection was performed on Ion Reporter™ versions 5.14 and 5.16 server system by the OCCRA - w2.5 - IR workflow. The quality control and variant calling analysis were performed on the Ion Reporter™ (IR) software package. Tertiary analysis and report generation was established using the GO Pathology Workbench (GenomOncology, Cleveland, OH).

### 2.5 Analytical validation

Analytical validation studies were carried out per guidelines from the Association for Molecular Pathology (AMP) and College of American Pathologists for the validation of Next-Generation Sequencing–Based Oncology Panels (Jennings et al., 2017). Details of the validation addressing STARD guidelines is presented in Supplementary Table S1.

#### 2.5.1 Specificity

Three Coriell HapMap DNA samples NA12878, NA18507, NA19240 and two normal colon and lung RNA samples (SeraCare Life Sciences, Milford, MA) were used to determine assay specificity by evaluating positive and negative variant calls of SNV/MNV, INDELs across all targeted hotspots and fusions covered by the assay. The hotspot and fusion design files (Thermo Scientific, Waltham, MA) were used to extract variants from VCF outputs followed by manual variant review.

#### 2.5.2 Sensitivity

Sensitivity was assessed using DNA and RNA from FFPE tissue, cell lines and contrived samples (Table 5). The true positive and false negative variants were determined by multiple commercial controls. Mean raw base calling accuracy was calculated for each of the samples with a target error rate <2%. The Coriell HapMap sample NA12878 is a well characterized benchmark sample for NGS validation studies. The AcroMetrix Oncology Hotspot Control (AOHC, Thermo Scientific, Waltham, MA) is a synthetic control consisting of 555 variants, with 198 covered by the OCCRA. The Seraseq Tri Level DNA Mutation Mix (SeraCare Life Sciences, Milford, MA) is a comprehensive synthetic control consisting of 40 mutations at target allele frequencies of 10, 7% and 4%, with 29 covered by the OCCRA. This control was sequenced 14 times during the validation to assess the assays’ ability of detecting variants at different allele frequencies. The Seraseq Fusion RNA Mix v4 (SeraCare Life Sciences, Milford, MA) is a reference standard containing a total of 16 fusions (14 gene fusions and 2 oncogenic isoforms). Fourteen of the 16 fusions are targeted by the OCCRA. The variant calling PPA (TP/(TP + FP) and PPV (TP/(TP + FN) was established for all variant types with IR default setting of ≥5% allele frequency (AF) for SNVs and INDELs, ≥4 copies for CNVs and ≥20 reads for fusion detection.

Limit of Detection (LOD) was determined for each variant type (SNV/MNV, INDELs, CNV and Fusions) using the contrived AOHC DNA Ladder, Seraseq Lung/Brain CNV and Seraseq RNA control titrated in a background of normal RNA (Placenta RNA Thermo Fisher). Limit of Input (LOI) was determined by diluting FFPE DNA and RNA in nuclease-free water. Nucleic acid concentration was measured using the Qubit™ dsDNA HS Assay Kit (Thermo Scientific, Waltham, MA) and Qubit™ RNA HS Assay Kit (Thermo Scientific, Waltham, MA) and two input concentrations (5ng and 1 ng) were used for downstream processing.

#### 2.5.3 Precision (repeatability and reproducibility)

Inter-assay repeatability was evaluated using three independent DNA and RNA libraries prepped from FFPE tissue and sequenced in triplicate on the same day, chips, and system. Two of the RNA samples were pooled from two different samples to increase the number of fusions assessed. To evaluate for inter-assay reproducibility, libraries from FFPE tissue and contrived controls were prepared for DNA (n = 5) and RNA (n = 4) and sequenced 2–5 times on multiple days, chips, and systems.

## 3 Results

### 3.1 Established thresholds and quality metrics

**FIGURE 1:**
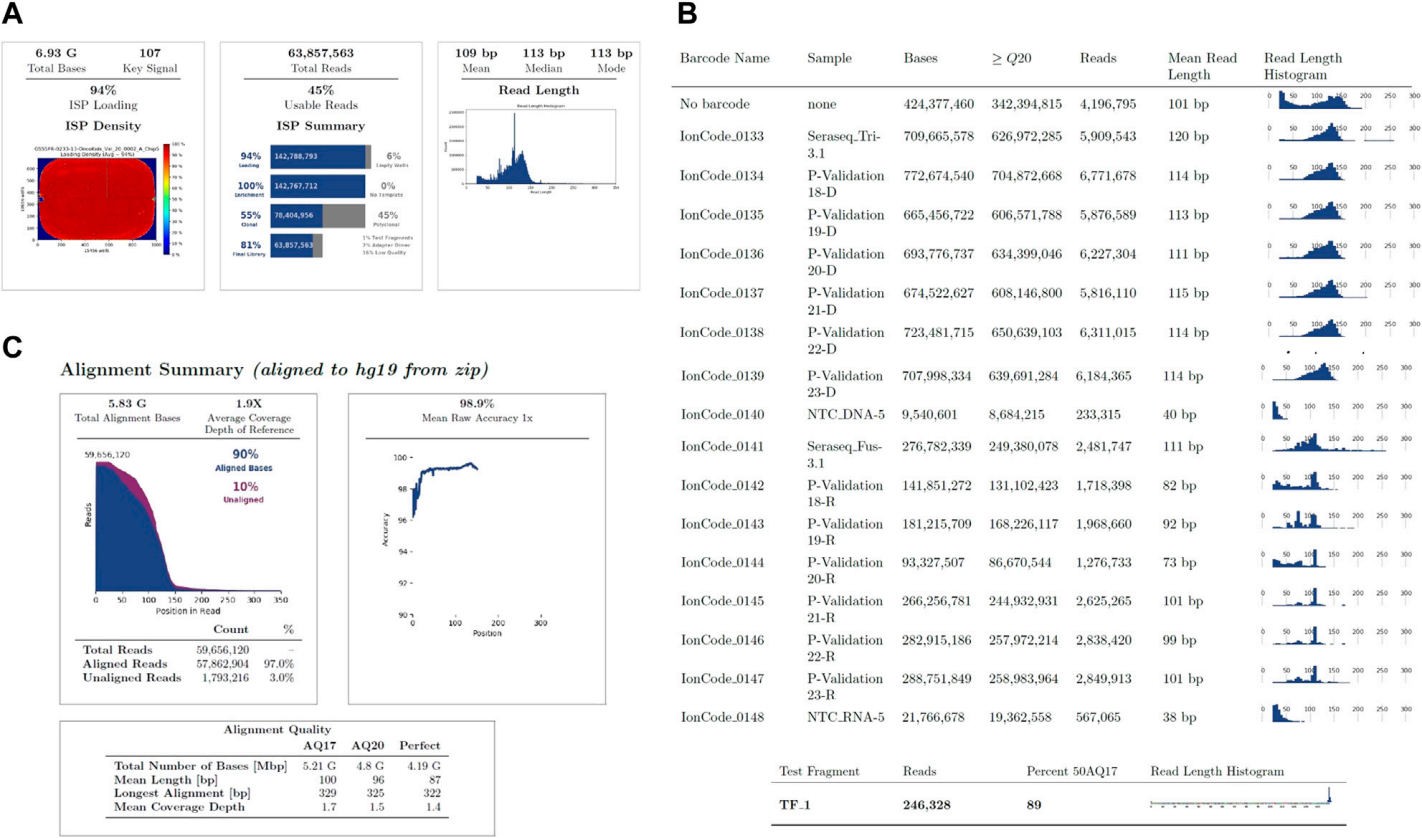
Run Summary Metrics obtained post sequencing. **(A)**. Summary of metrics across the chip with loading density which is expected to be at ≥85% (left panel), total number of reads being ≥60M and usable reads being ≥35% (middle panel) and the average read length evaluated (right panel). **(B)**. Run metrics for each sample on the chip. **(C)**. Sequence alignment summary.

The run metrics of the Ion Torrent Suite were used to determine quality control of sequencing runs which included base score, average sequencing depth, fusion panel control reads, minimum sequencing depth for variant calls, uniformity of coverage (ISP Loading), and strand bias of SNV and INDEL (Table 4; Figure 1). The thresholds of DNA mapped reads were 3M with mean depth ≥800x. The minimum mean read length was 75bp with uniformity ≥80% and mean raw accuracy ≥99%. The minimum RNA mapped reads was 20,000 with mean read length of 60bp.

**TABLE 4:** Quality metrics and thresholds.

Sample type	Mapped reads	Mean read length	Uniformity	Mean raw accuracy	Mean depth
DNA	3000000	75 bp	80%	99%	800
RNA	20000	60 bp	NA	NA	NA

### 3.2 Analytical accuracy

Analytical accuracy was established using the reference Coriell cell line NA12878 with a mean raw accuracy of 99.8% (Table 5). The Seraseq Tri-Level mix control targets variants at different allelic frequencies (4%, 7% and 10%) establishing the limit of detection to be ≥5% allele frequency (AF) for SNVs and INDELs since variants in the 3%–5% allele frequency range are detectable but display variable reproducibility. The minimum AF for small deletions (6–15 nt) was 3.4% and for small insertions (3-4 nt) was 3.8%. and SNVs were detected at 3.5% AF (Table 6). CNVs were detected at about 4.86–6.64 copies, depending on the cancer type (Table 7). All 14 fusions of the Seraseq Fusion v3 Mix control covered by the OCCRA, were detected at 43 reads (Table 8), establishing the cutoff to be 45 for clinical implementation. Automation of library prep resulted in the fusion detection cut-off being increased to 1,100 fusion spanning reads reducing the sensitivity for fusion detection, no impact was observed on the detection of DNA variants. SNVs, INDELs and fusions were able to be detected with 1 ng DNA and RNA input respectively. Gene amplifications were only detected with 5 ng of DNA (Table 9). Results from the AOHC established a PPA of 97% and a PPV of 100% (Table 10), with the combined PPA and PPV of all variants type at 97.2% and >99% with a 95% CI of 93.3%–99%, respectively (Table 11).

**TABLE 5:** Accuracy. Analytical Accuracy.

Sample	TP	TN	FP	FN	Analytical accuracy^a^
NA12878	125	1820	3	0	99.80%

^a^ Calculated using formula (TP + TN)/(TP + FP + TN + FN).

**TABLE 6:** Accuracy. The LOD of variant AF (SNVs and INDELs).

Gene ID	Cosmic ID	Identifier	HGVS nomenclature	Amino acid	Ladder 1 (%)	Ladder 2 (%)	Ladder 3 (%)
*EGFR*	COSM6225	Deletion	c.2236_2250del15	p.E746_A750delELREA	9.20%	5.70%	3.40%
*JAK2*	24,440	Deletion	c.1624_1629delAATGAA	p.N542_E543del	10.80%	5.70%	2.20%
*CEBPA*	18,099	Insertion	c.939_940insAAG	p.K313_V314insK	6.60%	3.80%	2.90%
*EGFR*	COSM12378	Insertion	c.2310_2311insGGT	p.D770_N771insG	7.10%	4.30%	1.50%
*NPM1*	17,559	Insertion	c.863_864insTCTG	p.W288fs*12	10.40%	4.50%	N/A
*ABL1*	12,560	Substitution	c.944C>T	p.T315I	5.70%	4.70%	2.20%
*AKT1*	COSM33765	Substitution	c.49G>A	p.E17K	9.10%	4.70%	1.50%
*BRAF*	COSM476	Substitution	c.1799T>A	p.V600E	11.00%	6.40%	2.90%
*CBL*	34,077	Substitution	c.1259G>A	p.R420Q	8.10%	5.30%	2.10%
*CBL*	34,055	Substitution	c.1139T>C	p.L380P	8.20%	5.40%	0.80%
*CSF3R*	1,737,962	Substitution	c.1853C>T	p.T618I	9.80%	5.20%	1.90%
*EGFR*	COSM6240	Substitution	c.2369C>T	p.T790M	7.40%	3.80%	1.50%
*EGFR*	COSM12979	Substitution	c.2573T>G	p.L858R	8.10%	5.30%	2.30%
*FLT3*	COSM783	Substitution	c.2503G>T	p.D835Y	10.40%	6.80%	2.90%
*IDH1*	COSM28747	Substitution	c.394C>T	p.R132C	8.20%	5.30%	2.50%
*JAK2*	COSM12600	Substitution	c.1849G>T	p.V617F	9.20%	4.30%	1.30%
*KIT*	COSM1314	Substitution	c.2447A>T	p.D816V	7.40%	4.60%	1.80%
*KRAS*	COSM521	Substitution	c.35G>A	p.G12D	5.60%	4.40%	2.80%
*MPL*	COSM18918	Substitution	c.1544G>T	p.W515L	7.70%	5.10%	1.40%
*PIK3CA*	COSM775	Substitution	c.3140A>G	p.H1047R	10.30%	5.90%	1.90%
*PIK3CA*	COSM763	Substitution	c.1633G>A	p.E545K	8.60%	5.20%	3.50%
*PIK3CA*	COSM760	Substitution	c.1624G>A	p.E542K	8.30%	5.20%	2.10%

**TABLE 7:** Accuracy. The LOD of CNV detection.

Gene	Expected detection	Detected copy number 1	Detected copy number 2	Detected copy number 3	Detected Copy Number T1
*MET*	Yes	6.94	6.56	6.95	—
*MYC*	Yes	5.35	5.01	5.26	—
*MDM2*	Yes	4.86	4.9	4.95	—
*ERBB2*	Yes	8.41	8.54	8.41	—
*MYCN*	Yes	11.46	11.53	10.62	6.94
*EGFR*	Yes	10.16	10.03	10.27	6.64
*MET*	Yes	10.62	10.49	10.85	6.87

**TABLE 8:** Accuracy. The LOD of fusion detection.

Fusion	5′fusion	5′exon	3′fusion	3′exon	OCCRA targeted	Detected	T1	T2	T3	T4
*CD74*-*ROS1*	*CD74*	6	*ROS1*	34	Yes	Yes	812	584	124	ND
*EGFR* vIII	*EGFR*	1	*EGFR*	8	Yes	Yes	1,822	ND	ND	ND
*EGFR*-*SEPT14*	*EGFR*	24	14-Sep	10	Yes	Yes	1,033	445	151	43
*EML4*-*ALK*	*EML4*	13	*ALK*	20	Yes	Yes	543	386	77	ND
*ETV6*-*NTRK3*	*ETV6*	5	*NTRK3*	15	Yes	Yes	4,243	2,186	846	119
*FGFR3*-*BAIAP2L1*	*FGFR3*	17	*BAIAP2L1*	2	Yes	Yes	2,302	2,116	271	ND
*FGFR3*-*TACC3*	*FGFR3*	17	*TACC3*	11	Yes	Yes	1,369	3,545	184	ND
*KIF5B-RET*	*KIF5B*	24	*RET*	11	Yes	Yes	2,828	2,821	611	ND
*LMNA*-*NTRK1*	*LMNA*	2	*NTRK1*	10	Yes	Yes	928	702	81	68
*MET* Exon 14 Skipping	*MET*	13	*MET*	15	Yes	Yes	^a^249 READ_COUNT ≤ 1,000			
*NCOA4*-*RET*	*NCOA4*	8	*RET*	12	Yes	Yes	258	121	63	ND
*SLC34A2*-*ROS1*	*SLC34A2*	4	*ROS1*	34	Yes	Yes	756	570	ND	ND
*SLC45A3*-*BRAF*	*SLC45A3*	1	*BRAF*	8	Yes	Yes	201	160	ND	ND
*TPM3*-*NTRK1*	*TPM3*	7	*NTRK1*	9	Yes	Yes	4,531	3,962	2,047	ND

^a^ Cutoff for MET, Exon 14 skipping is ≥ 1,000 fusion spanning reads.

**TABLE 9:** Accuracy. The LOI of DNA and RNA.

5 ng result	Depth at variant call/Fusion control reads	5 ng AF/CN/Fusion reads	1 ng result	Depth at variant call/Fusion control reads	1 ng AF/CN/Fusion reads
Detected	3,246	47.20%	Detected	1,507	45.30%
Detected	2,030	50.70%	Detected	958	51.70%
Detected	3,373	46.20%	Detected	1,734	48.50%
Detected	10,523	47.00%	Detected	6,318	45.40%
Detected	N/A	5.72	Not Detected	N/A	5.23
Detected	294,683	14,463	Detected	127,422	18,723
Detected	88,405	10,296	Detected	62,558	18,829
Detected	301,854	7,658	Detected	65,046	3,945

**TABLE 10:** Accuracy. PPA and PPV established by AcroMetrix Oncology Hotspot control.

Component	AOHC (n = 1)
True Positive Variants	192
False Positive Variants	0
False Negative Variants	6
Total Variants	198
PPA	97%
PPV	>99%

**TABLE 11:** Accuracy. Overall PPA and PPV of different variant types.

Component	SNP, MNP	INDEL	CNV	Fusion
Criteria	≥5% AF @ 100X Depth	≥5% AF @ 100X Depth	≥4 Copies	≥20 Reads
True Positive	62	18	7	55
False Positives	0	0	0	0
False Negatives	0	0	0	4
Min AF/CN/Reads	4.80%	5.20%	4.86	184
Max AF/CN/Read Counts	98.80%	66.10%	11.46	462,174
Average AF/CN/Read Counts	19.40%	23.00%	8.26	32,067
Min depth at variant call	738	1,074	NA	NA
Max depth at variant call	5,951	3,885	NA	NA
Average depth at variant call	2,781	2,561	NA	NA
PPA	>99%	>99%	>99%	93.20%
PPV	>99%	>99%	>99%	>99%

### 3.3 Specificity

A total of 3,640 negative variants were identified in both NA12878 and NA19240 samples with 772 INDELs and 2,869 SNVs/MNVs. Total 1820 negative variants were identified in NA18507 sample with 386 INDELs and 1,434 SNVs/MNVs. There were no positive variants detected across all hotspots, giving a specificity of ≥99% for all HapMap DNA samples (Table 12). Two normal colon and lung RNA samples were used to establish specificity of fusion detection of the assay. There was one false positive non-targeted fusion FHIT-TIRAP. F8T4 detected at 2,827 reads in one of the normal colon RNA replicates, resulting in the specificity greater than 99% in fusion detection (Table 13).

**TABLE 12:** Specificity. Analytical specificity of DNA samples.

Component	NA12878 (n = 2)	NA18507 (n = 1)	NA19240 (n = 2)
Positive Variants	0	0	0
Negative Variants	3,640	1820	3,640
Total Variants	3,640	1820	3,640
INDEL Pos	0	0	0
INDEL Neg	772	386	772
SNV, MNV Pos	0	0	0
SNV, MNV Neg	2,868	1,434	2,868
Specificity	>99%	>99%	>99%

**TABLE 13:** Specificity. Analytical specificity of RNA samples.

Component	Normal colon RNA (n = 2)	Normal lung RNA (n = 2)
Total Fusion Positive	^a^1	0
Total Fusion Negative	3,406	3,406
Total Fusions	3,406	3,406
Average Control Reads	176,592	72,522
Specificity	>99%	>99%

^a^ Positive non-targeted FHIT-TIRAP. F8T4 @ 2,827 reads.

### 3.4 Repeatability and reproducibility

A total of 39 true positive variants of SNV/MNV, INDEL, CNV and fusions were detected across the samples and all replicates, resulting in an overall combined variant repeatability of >99% (95% CI of 91.0%–100%) (Table 14). A total of 73 true positive variants of SNV/MNV, INDEL, CNV and fusions were detected in the combined samples and all replicates resulting in an overall combined variant reproducibility of >99% (Table 15).

**TABLE 14:** Repeatability and Reproducibility. Intra-assay Repeatability.

Component	SNV/MNV	INDEL	CNV	Fusion
Criteria Cutoff	≥5% AF @ 100X Depth	≥5% AF @ 100X Depth	≥4 Copies	≥20 Reads
True Positive	15	6	3	15
False Positive	0	0	0	0
False Negative	0	0	0	0
Repeatability	>99%	>99%	>99%	>99%

**TABLE 15:** Repeatability and Reproducibility. Inter-assay Reproducibility.

Component	SNV/MNV	INDEL	CNV	Fusion
Criteria Cutoff	≥5% AF @ 100X Depth	≥5% AF @ 100X Depth	≥4 Copies	≥20 Reads
True Positive	18	7	22	26
False Positive	0	0	0	0
False Negative	0	0	0	0
Reproducibility	>99%	>99%	>99%	>99%

## 4 Discussion

The present study describes the analytical validation and implementation of a pan cancer NGS panel CANSeq^TM^Kids for the detection of clinical actionable variants in childhood malignancies. Using a total of 65 samples, the study determined that the assay performed with greater than 99% accuracy, sensitivity, repeatability, and reproducibility, across different specimen types. Assay was optimized to use low input DNA (1–5 ng) and RNA (1 ng). Limit of detection of the assay was established to be ≥5% allele fraction for SNVs and INDELs, ≥4 copies for gene amplifications and 1,100 reads for gene fusions with automated library preparation. The study is presented in line with STARD (Standards for Reporting of Diagnostic Accuracy Studies) guidelines (Cohen et al., 2016), details are provided in Supplementary Table S1. The validated assay implemented for patient testing is listed on the National Institute of Health Genetic Test Registry (https://www.ncbi.nlm.nih.gov/gtr/labs/500088/), associated with the clinical test menu of the Precision Medicine Laboratory.

Targeted sequencing of a subset of genes is the most common test in clinical molecular diagnostic laboratories. However, given the various tumor types and molecular profiles of childhood malignancies, small gene panels that only covers genes of certain tumor type cannot satisfy the needs for appropriate disease management. The validation of the CANSeq^TM^Kids included over 30 childhood tumor types/subtypes (Table 2) and includes comprehensive screening of 230 unique genes known to be associated with childhood malignancies across FFPE, whole blood and bone marrow specimens. The CANSeq^TM^Kids evaluates both RNA and DNA for exonic hot spot regions of 86 genes, complete exonic regions of 44 genes, copy number of 28 genes and 91 fusion genes with variant types such as SNVs, INDELs, gene amplifications and gene fusions being detected. Overall, the assay covers a wide range of clinically actionable genes for a multitude of childhood tumor types and has greater than 99% accuracy, sensitivity, repeatability, and reproducibility with lower nucleic acid input amounts.

## Data Availability

The data presented in the study are deposited in the https://submit.ncbi.nlm.nih.gov/subs/sra/SUB12695707/overview repository, accession number SUB12695707.
